# The effect of the combined self-talk and mental imagery program on the badminton motor skills and self-confidence of youth beginner student-athletes

**DOI:** 10.1186/s40359-023-01073-x

**Published:** 2023-02-03

**Authors:** Yusuf Hidayat, Yunyun Yudiana, Burhan Hambali, Kuston Sultoni, Umit Dogan Ustun, Chatkamon Singnoy

**Affiliations:** 1 Faculty of Sport and Health Education, Universitas Pendidikan Indonesia, Bandung, Indonesia; 2 Sydney School of Education and Social Works, The University of Sydney, Sydney, Australia; 3 School of Physical Education and Sports, Mustafa Kemal University, Antakya, Turkey; 4 Faculty of Sport Science, Burapha University, Chon Buri, Thailand

**Keywords:** Psychological strategy, Badminton motor skill, Self-confidence, Mental imagery, Self-talk

## Abstract

**Background:**

The psychological strategies of self-talk (S.T.) and mental imagery (MI) have an essential role in training and sports performance, but their implementation, particularly in combination, is still limited. This study aimed to examine badminton motor skills (BMS) and self-confidence (S.C.) mastery after a psychological strategy intervention of S.T. and MI, which were integrated into the BMS training process in both independent and interactive functions. The S.T. strategy consisted of instructional (I-S.T.) and motivational (M-S.T.) functions, while the MI consisted of cognitive (C-MI) and motivational (M-MI) aspects.

**Methods:**

Eighty youth beginner badminton student-athletes aged 10–12 years (male = 40, female = 40) were divided through a disproportional stratified sampling into four experimental groups (EG: 2-function S.T. × 2-function MI) and one control group (CG). The intervention program lasted eight weeks (three meetings a week for a total of 24 meetings). The participants completed a fidelity check at each session. At the end of the intervention, they took a BMS test and completed a self-confidence scale.

**Results:**

The S.T. and MI strategies had a significant effect on BMS and S.C. mastery (independent and interaction functions) in multivariate and univariate ways; however, independently, they had no significant effect on S.C. The effect of I-S.T. > M-S.T. and the effect of C-MI > M-MI on BMS, but there was no difference in S.C. In the I-S.T. condition, the C-MI and M-MI strategies did not have a different interaction effect on BMS mastery, but the effect of M-MI > C-MI on S.C. In the M-S.T. condition, the effect of C-MI > M-MI on the BMS and S.C. mastery. In the C-MI condition, the I-S.T. and M-S.T. strategies did not have a different interaction effect on BMS mastery, but the effect of M-S.T. > I-ST on S.C. In the M-MI condition, the effect of I-S.T. > M-S.T. on the BMS and S.C. mastery.

**Conclusion:**

The results of this study contribute to the existing evidence on the effectiveness of S.T. and MI strategies in the motor skill mastery and psychological skill development of beginner student-athletes. Thus, S.T. and MI strategies can be adapted as psychological strategies that coaches and physical educators can use to improve beginner student-athlete learning, sports performance, and psychological skills.

## Introduction

Performance achievement can be gained in two core contexts, training and competition [1]. Peak performance when competing can be obtained using performance prospects that integrate the physical and mental aspects of sports skills through the right mindset to produce maximum performance achievement [2]. The unique characteristics and individual situations of each athlete need to be considered and are connected to sports psychology and its relevance in influencing the focus of athletes' performance in competition [2–4].

The quality and success of the sports training process is determined by its physical, technical, tactical, and psychological aspects. These four aspects contribute positively to the performance of athletes, both individually and as a team [5, 6]. Sports performance with correct movement techniques (sport skills) plays a very important role in the success of athletes from the early stages of training [7], including in badminton performance. Clear lob and high service are badminton motor skills (BMS) that should be first taught and mastered by beginner student-athletes. Every badminton player needs to master these two BMS because they form the basis for developing more complex strokes at the next learning stage, determine the overall movement in the game, and serve as an initial barometer for his or her ability to play badminton at the most basic level; to achieve high performance, a badminton athlete needs to effectively and efficiently master their BMS [8, 9].

To perform these two BMS well, an athlete needs not only physical and technical training but also psychological skill training or mental training programs. The results of a systematic review of mental training programs in racket sports show that the various techniques or strategies used in these programs have a positive influence on improving performance and mental skills [10]; two of the most frequently used psychological strategies are self-talk (S.T.) and mental imagery (MI) [11].

One type of mental or psychological skill that plays an important role in helping athletes achieve is self-confidence (S.C.) [12], which has been shown to influence behavior, attitudes, and sports achievement [13]. S.C. is a component of the fundamental skill that is needed to achieve success in sports [14] and is a key skill that is consistently demonstrated by successful elite athletes [15]. However, S.C. is “fragile” and is easily changed and influenced by other determinants [16]. Therefore, S.C. should be developed from an early age of training through various psychological strategy interventions.

As a psychological strategy, S.T. is a multidimensional phenomenon that is manifested in positive or negative, open or closed statements about oneself and has instructional and motivational functions [17]. S.T. can be used for a variety of motor tasks, participants, and contexts, such as for students in physical education classes [18], elite athletes [19], and beginner student-athletes [17, 20]. The S.T. strategy has instructional (I-S.T.) and motivational (M-S.T.) functions. I-S.T. consists of instructional specific, which is related to developing motor skills, and instructional general, which is connected to improving performance and playing strategies. M-ST consists of motivational arousal, motivational mastery, and motivational drive functions, which can be used to manage arousal level, increase attention, concentration, and S.C., and increase motivation and effort, respectively [21].

The S.T. strategy can be used on its own or in combination with other psychological skill training strategies [22]. This strategy has been shown to be effective in improving motor performance in various sports [23–25], increasing S.C. [26], self-efficacy [18, 27], and exercise motivation [17, 28], reducing anxiety [29], and managing attentional focus [30]. The results of the systematic review by Tod et al. [31] and a meta-analysis of the relationship between S.T. and sports performance [32] provide robust evidence on the effectiveness of S.T. strategy interventions in learning and sports performance, including its influence on BMS, which are the focus of this study. Regarding the effect of S.T. on S.C., among others, it has been proven that S.T. is effective in increasing S.C. and reducing anxiety for 72 young competitive tennis players [29] and helping to improve the performance and learning of tae-kwon-do skills, as well as improving psychological dimensions, including S.C. [20] and successfully increasing mastery of clear lob-BS and S.C.; the combination of I-S.T. and M-S.T. was proven successful in increasing mastery of clear lobs-BS and S.C. more than using only I-S.T. or M-S.T., and I-S.T. functions are more effectively used to improve clear lob-BS mastery [17].

As a multidimensional phenomenon, MI is defined as a representation of mental activity to imagine or recall an experience in mind using one or more sensory aspects [33] without the presence of an actual external stimulus. Conceptually, the MI strategy is divided into cognitive (C-MI) and motivational (M-MI) functions. C-MI can be separated into cognitive specific, for developing skills, and cognitive general, for developing strategies, while M-MI is divided into motivational specific, motivational general-arousal, and motivational general-mastery, which are helpful for achieving goals, managing tension, anxiety, and arousal, and developing S.C., mental toughness, and attention, respectively [34–36]. The cognitive and motivational functions of the MI strategy can be used at any time, in various situations, and for different purposes before, during, and after training or competition [37]. The MI strategy can be used alone [38] or in combination with other psychological skill training strategies in a single intervention program [39].

Conceptual studies and research results show that MI can facilitate learning and sports performance [40, 41] for various sports and participants, including college students [42], high school student-athletes [43], adult elite athletes [44], child elite athletes [45], and child beginner athletes [8, 46]. In addition, the MI strategy is also used to develop the psychological aspects of various movement tasks in sports [21], such as to increase motivation [47], S.C. [48], and self-efficacy [49] and reducing anxiety [48]. Several findings in experimental research indicate that the MI strategy is effectively used to increase the vividness of imagery, skiing performance, and S.C. [50], improve the S.C. of junior badminton players [51], C-MI and M-MI succeeded in increasing clear lob-BS and S.C. were higher and significant than those in the control group (CG), and there was a positive correlation between the clear lob-BS and S.C. mastery of 42 badminton beginner student-athletes [52].

Research on S.T. and MI strategies in sports activities has been widely carried out using various theoretical perspectives and methodological issues. However, these two strategies have generally been examined separately. Although the research results show a positive effect of S.T. and MI on sports performance and psychological aspects, some of the results of these studies are inconsistent (S.T.: [53]; MI: [38]). There are often confounding results between types and function, both between I-ST and M-S.T. as well as between C-MI and M-MI. I-S.T. conceptually functions to improve motor skills, performance, and playing strategies but empirically has also been found to be effective in improving M-S.T. functions, such as increasing motivation, attention, and S.C., reducing anxiety, managing arousal, and vice versa [18, 21, 23, 54]. Likewise, C-MI, a type of MI that conceptually functions to improve motor skills, performance, and playing strategies, has been empirically proven successful in increasing motivational aspects (M-MI function), such as managing arousal, reducing anxiety and increasing attention, S.C., motivation, mental toughness, and vice versa [47, 55].

Even the research on beginner student-athletes was not only inconsistent but also limited [S.T.: 23, MI: 56], especially when the two strategies were combined using a nomothetic design. Several studies have been conducted, including comparative research on the effect of a combination of S.T. and MI strategies (positive and negative) on the self-efficacy and dart-throwing skills of 95 students [39], on the dart-throwing mastery of athletes aged 12–16 years [57], and on self-efficacy development [58].

Research on the interaction function of S.T. and MI strategies in relation to mastery of BMS and S.C. is still limited, especially for badminton and youth beginner student-athlete groups. Some of these studies examined skilled athletes; for example, it was proven that the combination of S.T. and MI was more effective than either S.T. or MI in reducing state anxiety and increasing the S.C. and archery performance of 45 Malaysian national archery athletes [59]; it was also found that the combination of motivational-ST and MI, performed before serving, was beneficial for 33 skilled tennis players [60]. It was found that the MI intervention and the combination of MI and S.T. succeeded in increasing the percentage of service success and technical quality scores from pretest to posttest among novice tennis players. In addition, in the posttest, it was found that the score of technique quality and service speed in the MI and S.T. combination group was higher than the MI group and the control group [61]”.

In this study, each independent variable has two functional categories: the S.T. strategy function (I-S.T. and M-S.T.) and the MI strategy function (C-MI and M-MI). The theoretical justifications used to explain the effect of the S.T. strategy interaction with MI on BMS include the dual coding model [62] and the action language imagination model [63]. According to those models, the information needed to learn motor skills and sports performance comes from nonverbal and verbal information channel systems. Both channel systems can be inherently linked in the learning or training process. Meanwhile, the effect of the S.T. strategy on S.C. is abstracted using self-efficacy theory [64], which places past performance accomplishments, vicarious experiences, and verbal persuasion as important antecedents that can affect individual self-efficacy perceptions.

The implementation of a combination of S.T. and MI strategy functions in sports activities is urgent because, in addition to the inconsistency of the previous study results, there is a gap between understanding the important role of S.T. and MI strategies as “das-sollen-what should be” and its implementation in the field as “das sein-what is happening”. S.T. and MI strategies are rarely applied in badminton coaching, especially for youth beginner student-athletes, which constitute the largest age group in badminton lessons. Developing BMS, as a type of skill that beginner student-athletes must first master, becomes the basis for the development of other more complex basic skills and varies with the development of physical abilities, tactical skills, and psychological skills, including S.C., in a multidirectional manner and becomes a foundation skill in the psychological skill training process. The mastery of BMS is a manifestation of changes in training behavior in the psychomotor domain. The changes that occur are associations between functional units (mind, brain, and body), sociological and biophysical systems (environment), and student-athlete psychological attributes such as motivation and S.C. Increasing one’s mastery of BMS has a motivational effect on S.C.; conversely, an increase in S.C. stimulates increased mastery of BMS. The increase in both demonstrates the interaction of a cyclical reciprocal or bidirectional relationship.

In this constellation, this research aimed to test the level of BMS and S.C. mastery based on the S.T. (I-S.T., M-S.T.) and MI (C-MI, M-MI) strategy intervention, examining both the partial function (main effect) and the interactive function (interaction effect) integrated into two BMS training processes, compared to the results without S.T. and MI intervention (only performing two BMS mastery training sessions) for youth beginner student-athletes aged 10–12 years at a badminton school in Bandung City. The age range of 10–12 years is an early age for training. Starting at this age is considered not only one of the most basic strategies for world achievement but also very crucial because training at an early age can be ideal (various physical and social psychological aspects are open to learning in this age period), valuable for fostering and developing growth, and prospective in terms of age and quantity [65, 66].

We hypothesized the following: S.T. strategy has a significant influence on BMS and S.C. jointly (Hypothesis 1); S.T. strategy has a significant effect on BMS separately (Hypothesis 1a); S.T. strategy has a significant effect on S.C. separately (Hypothesis 1b); MI strategy has a significant effect on BMS and S.C. jointly (Hypothesis 2); MI strategy has a significant effect on BMS separately (Hypothesis 2a); MI strategy has a significant effect on S.C. separately (Hypothesis 2b); S.T. and MI strategies have an interaction effect on BMS and S.C. jointly (Hypothesis 3); S.T. and MI strategies have an interaction effect on BMS separately (Hypothesis 3a); S.T. and MI strategies have an interaction effect on S.C. separately (Hypothesis 3b); the BMS mastery of student-athletes who were treated with I-S.T. was higher than those who were treated with M-S.T. (Hypothesis 4); and the BMS mastery of student-athletes who were treated with C-MI was higher than that of those who were treated with M-MI (Hypothesis 5).

## Methods

### Design

This study has two independent variables, namely, S.T. (I-S.T., M-S.T.) and MI (C-MI, M-MI) strategies, and two dependent variables, namely, BMS and S.C. According to each independent variable category, there are four experimental groups, namely, the combination of I-S.T./C-MI (EG-1), I-S.T./M-MI (EG-2), M-S.T./C-MI (EG-3), and M-S.T./M-MI (EG-4). Each group was manipulated to examine its effect on the dependent variable separately and jointly. A 2 × 2 factorial design was used. The research hypothesis was tested with posttest measures on the dependent variables BMS and S.C., as was conducted by [17, 67, 68]. The pretest measurement of BMS and S.C. were not carried out because all the participants were beginner student-athletes who did not yet have BMS and were assigned randomly into five groups [68], and the S.C. scale was specially developed in the context of badminton training.

### Participants

**Fig. 1:**
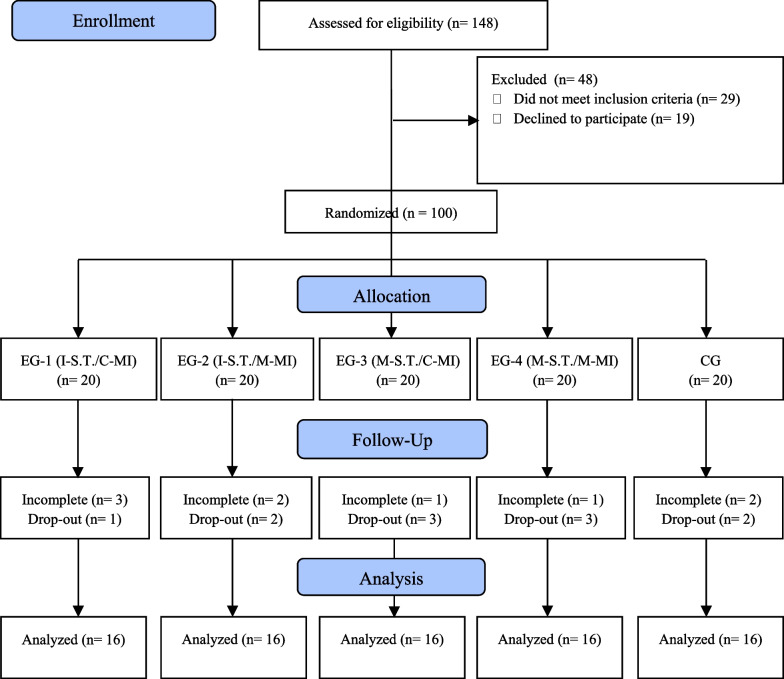
Participant recruitment and flow through the study

Youth beginner student-athletes at Badminton Schools in Bandung City participated in this study. The inclusion criteria were as follows: (1) male and female youth beginner athletes aged 10–12 years, (2) registered as student-athletes at badminton schools in Bandung City, and (3) signed and returned the informed consent. A CONSORT study flow diagram is shown in Fig. 1. A total of 148 youth beginner student-athletes, 78 male and 70 female, met the inclusion criteria. Furthermore, to select and assign participants to the experimental and control groups, the following steps were taken: (1) all parents of the participants provided informed consent; according to the informed consent forms, 129 youth beginner student-athletes, 62 male and 67 female, were willing to be involved in intervention activities; (2) 100 participants, consisting of 50 male and 50 female youth beginner student-athletes were assigned to be involved in the intervention through a disproportionate stratified sampling technique [68]; (3) all randomly selected participants to be matched (blocked random assignment) were assigned into five groups, each group consisting of 20 people, 10 male and 10 female youth beginner student-athletes; and (4) each group of participants was assigned randomly to all treatment combinations so that each group of participants received one treatment combination, while one group of participants became the control group.

The research team conducted this randomization process in a blinded manner to keep the participants from knowing their participation in the study as part of either the experimental or control group. Furthermore, based on the accumulation of attendance during the intervention process, the data from only 16 people in each group were ultimately analyzed. The data of the rest of the participants were not analyzed because they did not meet the required number of attendances during the experimental process. Therefore, the number of participants included in the data analysis was 80 people, aged 10–12 years (M_age_ = 11.4, SD_age_ = .52), consisting of 40 male beginner-student athletes (M_age_ = 11.3, SD_age_ = .58) and 40 female beginner athletes (M_age_ = 11.5, SD_age_ = .48) (see Table 1).

**Table 1:** Descriptive statistics of subject demographic characteristics in each group

Group	Demographic characteristic variables					
	Age		Height		Weight	
	M	SD	M	SD	M	SD
EG-1 (NG = 16); N_Ma_ = 8, N_Fe_ = 8	11.1	0.78	137.54	5.7	30.48	4.75
EG-2 (NG = 16); N_Ma_ = 8, N_Fe_ = 8	11.5	0.58	141.38	9.06	36.94	11.67
EG-3 (NG = 16); N_Ma_ = 8, N_Fe_ = 8	11.5	0.37	143.67	5.14	35.88	7.92
EG-4 (NG = 16); N_Ma_ = 8, N_Fe_ = 8	11.5	0.57	142.73	7.81	36.61	12.54
CG-5 (NG = 16); N_Ma_ = 8, N_Fe_ = 8	11.6	0.31	139.31	6.18	34.31	6.23
Total subject = 80	11.4	0.52	140.93	6.78	34.84	8.62

M, Mean; SD, Standard deviation; EG-1–EG-4, Experimental groups 1–4; CG, Control group; NG, Number of participants in a group; N_Ma_, Number of male participants in a group; N_Fe_, Number of female participants in a group

### Measures

#### Badminton motor skill test (BMS test)

The BMS test refers to the learning outcome of the badminton basic skill test [17], which measures participants’ ability to perform clear lob and high service-BS tests on the outcome-based test (OBT) aspect and process-based test (PBT) aspect in the preparation and hitting phase dimensions. The analysis results of the internal consistency estimation produced a correlation coefficient value of Cronbach’s alpha of .92. Person Product Moment was used to measure interclass reliability, and r = .84 to .94 for OBT and .80 to .85 for PBT. A correlation coefficient of .72 was obtained from the Person Product Moment correlation analysis for criterion-related validity of OBT using a concurrent validity approach and the criteria for the assessment results of five expert judgment panels on the BMS movement process. From the factorial validity process of the PBT test using confirmatory factor analysis, the factor loading (FL) values obtained from all indicators were between .60 and .84.

#### Self confidence scale (SCS)

The SCS was used to measure the level of student-athletes’ confidence in their ability to succeed in performing the BMS after they completed the intervention program. The SCS was developed based on the S.C. multidimensional model in sports [69], consisting of the cognitive efficiency (CE-D), physical skills and training (PST-D), and resilience dimensions (R-D). A trial with 254 respondents obtained an estimated index of internal consistency reliability, as the Cronbach’s alpha of SCS = .91 (48 items), CE-D = .76 (18 items), PST-D = .71 (12 items), and R-D = .78 (18 items). The estimated factorial validity using confirmatory factor analysis obtained 32 valid items (FL = .52 to .82), consisting of 12 CE-D items (FL = .67 to .78), 8 PST-D items (FL = .57 to .81), and 12 R-D items (FL = .52 to .78).

### Fidelity check

The S.T. fidelity check was carried out to ensure that the participants used S.T. during practice in accordance with what they learned about understanding the indicators of how to use S.T., the level and frequency of S.T. use, the most frequently used S.T., the benefits of using S.T., and the reasons for not using S.T., including no reasons, not sure about the benefits, and not knowing how to do it [17]. The MI fidelity check was used to ensure the implementation of MI training on understanding the indicators of the process (how to do), content, perspective, and benefits of MI [70, 71]. Fidelity checks were only given to the experimental group participants, according to the type of assigned S.T. and MI. An example of a question for a S.T. fidelity check is as follows: Question: “Do you understand how to use self-talk in today’s training?”, Answer: understand, understand a little, do not understand”. S.T. and MI fidelity checks were given immediately after every intervention meeting and consisted of a set of questions that the participants had to answer.

### Intervention and procedure

#### Research stages

The research was carried out in the following three stages: preparation, implementation, and evaluation. The preparatory stage consisted of preparing and validating S.T. and MI training modules and programs, as well as teaching S.T. and MI to coaches through workshops and practical training [9]. The implementation stage consisted of the substages of education, training, and evaluation. The education substage contained program socialization activities for participants in two class meetings, and each meeting consisted of 120 min [8]. The training substage was an intervention implementation activity for 11 weeks (24 meetings, three times per week, 130 min for each meeting) from July to September 2021. Before the intervention, all participants reported their demographic variables (age, weight, and height), and then the intervention was implemented in the opening stage (30 min: attendance, explanation of training objectives, demonstration of BMS and S.T. instructions, external MI training, and warm-up), the core training stage (80 min: BMS acquisition, game training, and use of S.T. in every training session, except the CG), and the closing stage (20 min: internal MI training, S.T. and MI fidelity check, and cooling down activities). According to the combination of S.T., MI, and CG function categories, there were five groups of treatment combinations, namely, (1) EG-1: I-S.T./C-MI and mastery of BMS, (2) EG-2: I-S.T./M-MI and mastery of BMS, (3) EG-3: M-S.T./C-MI and mastery of BMS, (4) EG-4: M-S.T./M-MI and mastery of BMS, and (5) CG: mastery of BMS. The evaluation stage consisted of measuring the BMS and filling in the SCS immediately after the 24th meeting.

#### Stages and approach of intervention

S.T. strategy was incorporated into the intervention program based on the results from a workshop with 16 basic-level badminton coaches and consisted of the following stages: (1) elaborating the motor skills to be mastered into specific movement parts, (2) developing an applicative framework, (3) explaining the S.T. strategy to the participants, (4) giving assistance and social feedback during the intervention, and (5) giving fidelity checks [9]. The four stages of the results of this workshop, based on the ideas and research results of Hatzigeorgiadis et al. [72] on ways or strategies for effectively implementing S. T interventions, are compiled in the acronym S.T.-IMPACT (Identify what you want to achieve, Match S.T. to needs, Practice different cues with consistency, Ascertain which cues work best for you, Create specific S.T. plans, dan Train S.T. plans to perfection). The applicable framework of the “four Ws: where when, what, and why” was used [9, 21], while the intervention type was assigned-S.T. [24, 73]. S.T. was used in the BMS training process (where) before, during, and after performing the movement (when). The cue nature of S.T. was positive, singular or specific or general phrases, using the first person singular (what) and based on the I-S.T. and M-S.T. functions (why). The specific S.T. cues were selected and determined based on the workshop with 16 basic-level badminton coaches. The workshop process was carried out for two days through four stages of the process (experience formation, reflection, concept formation, and concept testing). The product is an applicative concept of instructional and motivational S.T. and its function and use in the training process [9]. The I-S.T. cues for clear lob-BS were as follows: ready, see the shuttlecock, cross steps, behind the shuttlecock, open-shoulders, front-ears, hit, high-straight, whip’s end, cross swing, and ready again. Meanwhile, in the high service-BS, the cues were as follows: ready, back-maximum, front swing, hits, strong-whip’s end, cross swing, and ready again. The M-S.T. cues were as follows: focus, yes, I can, ready, readier, calm, inhaling a deep breath, good, staying motivated, keep trying, stronger, harder, strong-high to the back, show, and do your best [9, 17]. The S.T. cue was used serially. The sequence of using the I-S.T. cues was based on the parts of the movement being taught. Meanwhile, one M-S.T. cue was given at each meeting. At the following meeting, one more cue was added, and so on until the 12th meeting, and vice versa, for the 13th to 24th meetings.

The MI program in this study was based on the MI Intervention Education and Training Program, in the form of workshops and coaching practice training for 20 basic-level badminton coaches and 56 youth beginner badminton students [71]. In accordance with the program, the implementation of the MI strategy refers to the following process stages:

- Developing an applicative framework consisting of the Four “Ws” framework: where when, what, why [37, 74], who, and how. MI was given in practice situations (where), before and after practice (when), maximum 10 min for each MI training session, in positive MI, on the vividness and controllability dimensions, for both visual and kinesthetic MI types (what), and based on cognitive and motivational functions (why), given to youth beginner student-athletes aged 10–12 years (who), and given through tape recorder media and using keywords as markers of imagined objects according to S.T. (how) guidelines.
- The method used for writing MI scripts is the layering system method [75], a method of modifying the script that is arranged in stages to suit the characteristics of the participants (youth beginner student-athletes), the performance environment (practice situation), and the movement tasks required. will do (description and cue). The MI script format is divided into an introductory section (breathing regulation activities, relaxation exercises, and basic imagery exercises using all MI modalities), the main section (description of movement skills and situation descriptions related to mastery of movement skills with emphasis on keywords or MI-cue as markers, according to the type of motor skill), and the closing section (breathing regulation and strengthening activities to ensure that participants have performed MI exercises correctly).
- Integrating the applicative framework into the overall structure of the exercise program, starting from the opening section (attendance, training objectives, observation, S.T.-strategy, MI training, warming-up), core training (basic skill training, game play training, S.T.-training), and closing section (MI training, fidelity check, review, and cooling down);
- Communicate the MI program to student-athletes according to the conceptual framework to be used;
- In each MI training session, participants were in a relaxed state, in a supine or anatomically sitting position on the badminton court and started with basic MI training. At the end of each meeting, each student-athlete completed the S.T. and MI fidelity check sheets to determine the consistency of their use by each student-athlete during the training process.

### Statistical analysis

The data were analyzed using two-way MANOVA to test the effect of two independent variables simultaneously and separately on the variation of the two dependent variables, both multivariate and univariate. It is assumed that the two dependent variables had a strong theoretical basis for multivariate analysis. The differences between the EG and CG were analyzed using one-way MANOVA.

## Results

### Preliminary analysis

Preliminary analysis was used to determine differences among groups in the demographic variables of age, weight, and height. This analysis was conducted to ensure that the significance of differences in the dependent variable was not caused by variations in the differences in the three demographic variables but by variations in treatments.

Table 2 shows the results of one-way ANOVA for the demographic variables. The finding showed that there was no significant difference in age (F(4.75) = 1.57, *p* = .19) or in the weight (F(4.75) = 2.02 = .10) or height variables (F(4.75) = 1.79, *p* = .14).

**Table 2:** Results of one-way ANOVA differences in demographic variables among groups

Demographic Variable	df	F	Sig
Age	(4,75)	1.57	.19
Height	(4,75)	1.79	.14
Weight	(4,75)	2.02	.10

*p < .05; **p < .01

### Main analysis

#### Statistics description

The statistical description in Table 3 presents the mean and standard deviation scores of participants’ badminton motor skills and self-confidence after the intervention. Furthermore, the MANOVA test was conducted, the assumption test of MANOVA was carried out using the Kolmogorov‒Smirnov normality test, as well as the homogeneity test using the Levene statistic and the M Box test covariance matrix [76, 77]. Overall, the results of the analysis of the assumption test show that they meet the criteria for the MANOVA.

**Table 3:** The results of statistic descriptive measurement (means and standard deviations)

Dependent variable	S.T	MI	N	Mean	SD	Dependent variable	S.T	MI	N	Mean	SD
BMS	I-S.T./C-MI (EG-1)		16	38.45	7.73	S.C	I-S.T./C-MI (EG-1)		16	77.81	4.92
	I-S.T./M-MI (EG-2)		16	38.21	4.67		I-S.T./M-MI (EG-2)		16	80.31	3.44
	Total		32	38.33	6.28		Total		32	79.06	4.36
	M-S.T./C-MI (EG-3)		16	37.61	6.09		M-S.T./C-MI (EG-3)		16	81.19	2.48
	M-S.T./M-MI (EG-4)		16	28.80	7.99		M-S.T./M-MI (EG-4)		16	77.13	3.24
	Total		32	33.20	8.30		Total		32	79.16	3.51
	Total	C-MI	32	38.03	6.86		Total	C-MI	32	79.50	4.20
		M-MI	32	33.50	8.02			M-MI	32	78.72	3.67
		Total	64	35.77	7.75			Total	64	79.11	3.93
	CG		16	28.99	9.58		CG		16	74.94	2.05

N, Number of participants; SD, Standard deviation; EG, Experimental group; CG, Control group

#### Multivariate and univariate significance test of between-subject effects

**Fig. 2:**
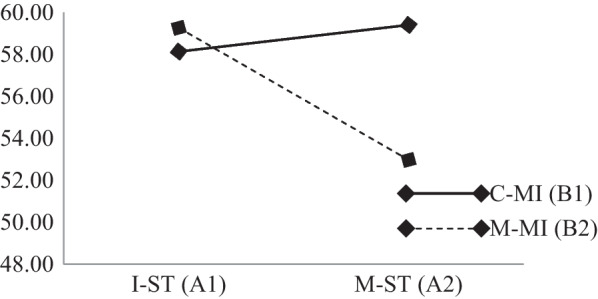
Effect of multivariate interaction between S.T. and MI on BMS and S.C

Based on the results of the multivariate test using Pillai's trace test, it was proven that S.T. had a significant effect on BMS and S.C. mastery (F (3.58) = 4.20, *p* = .01 < .05, Partial Eta Square (η^2^) = .18), on MI (F (3.58) = 3.10, *p* = .03 < .05, (η^2^) = .14), and on the interaction of S.T. and MI (F(3, 58) = 4.66, *p* = .01 < .05, (η^2^) = .19) (see Fig. 2).

**Fig. 3:**
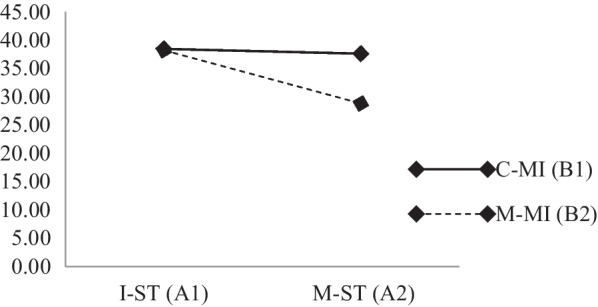
Effect of univariate interaction between S.T. and MI on BMS

**Fig. 4:**
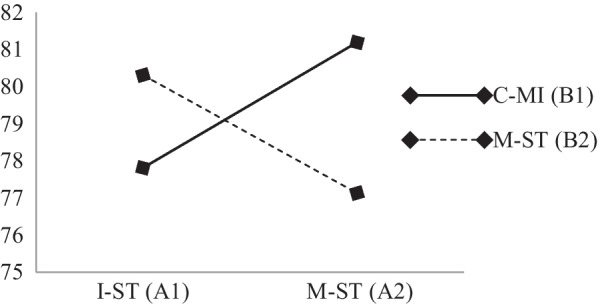
Effect of univariate interaction between S.T. and MI on S.C

According to the results of the univariate test, (1) S.T. had a significant effect on BMS mastery (F = 9.21, *p* = .00 < .05, (η^2^) = .13) but had no effect on S.C. (F = .01, *p* = . 92 > .05, (η^2^) = .00), (2) MI had a significant effect on BMS mastery (F = 7.19, *p* = .01 < .05, (η^2^) = .11) but had no effect on S.C. (F = .74, *p* = .39 > .05, (η^2^) = .01), and (3) the combination of S.T. and MI had a significant interactive effect on BMS mastery (F = 6.44, *p* = .01, (η^2^) = .10) and S.C. mastery (F = 13.08, *p* = .00, (η^2^) = .18) (see Figs. 3, 4).

#### Pairwise comparisons significance test

Based on the results of the pairwise comparison test (see Table 4), it was proven that (1) I-S.T. had a more significant effect than M-S.T. on BMS mastery (*p* = .00 < .05), but there was no difference in the effect on S.C. (*p* = .92 > .05) and (2) C-MI had a more significant effect than M-MI on BMS mastery (*p* = .01 < .05), but there was no difference in the effect on S.C. (*p* = .39 > .05).

**Table 4:** The results of the pairwise comparison analysis of independent variable categories

Pairwise comparison	Mean difference	Standard deviation	Conclusion
I-S.T.: M-S.T. on BMS	5.12 (− 5.12)	1.69	(*p* = .00 < .05)**
I-S.T.: M-S.T. on S.C	− .09 (.09)	.91	(*p* = .92 > .05)
C-MI: M-MI on BMS	4.53 (− 4.53)	1.69	(*p* = .01 < .05)**
C-MI: M-MI on S.C	− .78 (− .78)	.91	(*p* = .39 > .05)

*p < .05; **p < .01

The results of the pair interaction test between S.T. and MI (see Table 5) are as follows: (1) the effect of I-S.T. > M-S.T. on the BMS and S.C. mastery in the M-MI condition, but there was no difference in the interaction effect on BMS mastery in the C-MI condition. On the other hand, the effect of M- S.T. > I-S.T. on S.C. in the C-MI condition. (2) In the M- S.T. condition, the effect of C-MI > M-MI on the BMS and S.C. mastery, but there was no difference in the interaction effect on BMS mastery in the I-S.T. condition. On the other hand, the effect of M-MI > C-MI on S.C. in the I-S.T. condition.

**Table 5:** Results of the comparison analysis of the pair interaction between S.T. and MI

Pair interaction comparison	Mean	Independent variable	Conclusion
Comparison between I-S.T. and M-S.T. on C-MI (I-S.T./C-MI: M-ST/C-MI)	38.45:37.61	BMS	I-S.T./C-MI ≠ M-S.T./C-MI (Not significant)
Comparison between I-S.T. and M-S.T. on M-MI (I-S.T./M-MI: M-ST/M-MI)	38.21:28.80	BMS	I-S.T./M-MI > M-S.T./M-MI (significant)
Comparison between I-S.T. and M-S.T. on C-MI (I-S.T./C-MI: M-S.T./C-MI)	77.81:81.81	S.C	I-S.T./C-MI < M-S.T./C-MI (significant)
Comparison between I-S.T. and M-S.T. on M-MI (I-S.T./M-MI: M-S.T./M-MI)	80.31:77.13	S.C	I-S.T./M-MI > M-S.T./M-MI (significant)
Comparison between C-MI and M-MI on I-S.T. (I-S.T./C-MI: I-S.T./M-MI)	38.45:38.21	BMS	I-S.T./C-MI ≠ I-S.T./M-MI (Not significant)
Comparison between C-MI and M-MI on M-S.T. (M-S.T./C-MI: M-S.T./M-MI)	37.61:28.80	BMS	M-S.T./C-MI > M-S.T./M-MI (significant)
Comparison between C-MI and M-MI on I-S.T. (I-S.T./C-MI: I-S.T./M-MI)	77.81:80.31	S.C	I-S.T./C-MI < I-S.T./M-MI (significant)
Comparison between C-MI and M-MI on M-S.T. (M-S.T./C-MI: M-S.T./M-MI)	81.19:77.13	S.C	M-S.T./C-MI > M-S.T./M-MI (significant)

#### The comparison between the EG and the CG

The results of multivariate test analysis using Wilks' Lambda showed that there were differences in the effect between groups on BMS and S.C. jointly (F(12,193) = 4.44, *p* = .00 < .05, (η^2^) = .19), also separately to BMS (F(4,408) = 7.43, *p* = .00 < .05, (η^2^) = .28) and S.C. (F(4,101) = 8.90, *p* = .00 < .05, (η^2^) = .32). The comparison between the EG and CG is presented in Table 6.

**Table 6:** Results of comparative analysis between the CG and EG

Dependent variable	Control group (CG) and experimental group (EG)		Mean difference (I–J)	Std. error	Sig.^a^	95% Confidence interval for difference^a^	
						Lower bound	Upper bound
BMS	CG	EG-1	− 9.46	2.62	.00**	− 14.67	− 4.24
		EG-2	− 9.22	2.62	.00**	− 14.43	− 4.00
		EG-3	− 8.62	2.62	.00**	− 13.84	− 3.40
		EG-4	0.20	2.62	.94	− 5.02	5.41
S.C	CG	EG-1	− 2.88	1.19	.02*	− 5.25	− 0.50
		EG-2	− 5.38	1.19	.00**	− 7.75	− 3.00
		EG-3	− 6.25	1.19	.00**	− 8.63	− 3.87
		EG-4	− 2.19	1.19	.07	− 4.56	0.19

*p < .05; **p < .01

Sig.^a^ = *p*-value

The results of the analysis in Table 6 show that in general, all EGs had a higher and more significant effect than CG on both BMS and SC, except for a comparison between CG and EG-4 (A2B2 or M-ST/M-MI), which proves that there is no difference, both in BMS (α = .05 (.94 > .05) and in SC. (.07 > .05).

### Fidelity check

#### S.T. fidelity check

Based on the results of the S.T. fidelity check, the participant responses regarding the use of S.T. were as follows: (1) 85.40% understood how to use S.T.; (2) S.T. was used during the exercise by 66.20% of the respondents, sometimes by 38.60% of the respondents, and never by 5.20% of the respondents; (3) the most frequently used S.T. were ready again (95.60% = clear lob-BS) and cross swing (98.43% = high service-BS); (4) for S.T. usage frequency, 70.40% of the participants responded “often”, 29.60% of the participants responded “sometimes”, and 10.00% of the participants responded almost never, (5) 81.3% reported that I-S.T. helped BMS mastery, (6) 77.6% reported that M-S.T. helped increase morale, and 71.3% reported that it helped improve S.C.; and (7) the reasons for not using S.T. included 6.30% had no reason, 62.35% were unsure of its benefits, and 31.35% did not know how to do it.

#### MI fidelity check

Participant responses based on the MI fidelity check results were as follows: (1) 89.20% understood how to perform MI; (2) 86.10% could clearly imagine the movement to be studied; (3) 78.40% could imagine the movement shown by the model correctly; (4) 79.20% could clearly imagine the movements they had learned; (5) 81.50% used MI to correctly imagine their own movements; (6) 86.30% felt the benefits of MI in mastering BMS; and (7) 70.70% felt the benefits of MI in increasing S.C.

## Discussion

### S.T. main effect

The purpose of this study was to examine the effect of the psychological strategies of S.T. (I-S.T., M-S.T.) and MI (C-MI, M-MI) on BMS and S.C. mastery either separately (main effect) or jointly (interaction effect). In general, it was found that the S.T. and MI strategies had a significant effect on the BMS and S.C. mastery, although that they did not separately affect S.C.

Evidently, the S.T. strategy had a significant effect on the BMS and S.C. mastery, regardless of the MI condition (Hypothesis 1 was supported). This finding complements the empirical evidence that S.T. has a simultaneous divergent function in facilitating learning, sports performance, and psychological skill development [31, 32, 43], especially for beginner student-athletes when they are learning new motor skills [17, 18]. The results of this study also describe the effectiveness of the S.T. function in relation to simultaneous mastery of BMS and S.C. There is a coherence among the S.T. cues used, while the function and the outcome of S.T. intervention are referred to as “meaningful S.T.”. Theoretically, the results of this study explain the following: first, S.T. is a cognitive strategy that serves to represent the planned movement program in the actual movement process [78]; second, S.T., in the rule-governed behavior concept, is an antecedent in a statement of a rule that functions to regulate behavior to achieve goals. In this concept constellation, the incorporation of S.T. into the process of mastering BMS is essentially a manifestation of the rule-governed behavior concept [79]. Third, the process of incorporating the S.T. function into mastering BMS is a process of activating the antecedents of self-efficacy, namely, past performance accomplishments, vicarious experience, and verbal persuasion [80]. These three antecedents function as social feedback providing information about correct movements, wrong movements, and ways to correct them, as well as direct and accumulative motivational implications on the psychological aspect development [81], including S.C. Fourth, to explain the mechanism of S.T. in relation to performance and S.C., Galanis et al. [82] developed a model that differentiates the S.T. mechanism into an attentional perspective and a motivational perspective (a prospective model of S.T. mechanism). In this model, S.C. is explained through a motivational perspective, an S.T. mechanism perspective that is related to the motivational interpretation of the influence of S.T. on the cognitive, affective, and behavioral aspects of motivation. This model also includes theoretical constructs and perspectives that can both be related to the study of S.T. mechanisms in their effects on self-efficacy, S.C. and anxiety, effort and persistence.

Separately, the S.T. strategy had a significant effect on BMS mastery (Hypothesis 1a was supported). The results of this study strengthen the evidence that S.T. is very effective as a psychological strategy to facilitate learning and improve performance in motor and sport settings [82]. In its function as a cognitive strategy, the incorporation of S.T. into BMS mastery served as a “specific trigger” that greatly helped beginner student-athletes focus their attention on the key elements of movement [20, 26]. In this constellation, training is the most powerful moderator variable affecting the effectiveness of S.T. [83] and plays a crucial role not only because it can strengthen the association between the S.T. cue used and the success of the movement to be performed but also because it can increase the internal and external attention of the beginner student-athletes to the key elements of the movement. Thus, the S.T. strategy is effectively used in BMS mastery because S.T. can help student-athletes regulate their learning behavior or training to achieve goals by increasing internal and external attentional focus on key elements of movement, as well as social feedback as a source of information on learning progress and the mobilizer of the learning efforts of student-athletes.

The results of this study were strengthened by the results of the S.T. fidelity check. In general, the indicators that were measured showed a positive level of S.T. implementation, although there were still participants who had not used it consistently, mainly because they were still unsure of its benefits. Furthermore, the findings on the simple effect interaction test show that the effect of I-S.T. on BMS and S.C. is more notable than that of M-S.T. combined with M-MI. However, the effect of M-S.T. on S.C. is more significant than that of I-S.T. when combined with C-MI. Therefore, the use of I-S.T. and M-S.T. is more effective in the process of mastering motor skills when they are combined with M-MI and C-MI, respectively.

### MI main effect

Based on the results of the multivariate test, it was found that the MI strategy had a significant effect on the BMS and S.C. mastery, regardless of the S.T. condition (Hypothesis 2 was supported). The results of this study are relevant to several conceptual studies and the results of previous research on movement learning and sports performance domains [40, 41], especially for young child student-athletes [8, 46, 84, 85], including the development of various psychological skills, such as improving S.C. [48], motivation [47], and self-efficacy [49] and reducing anxiety [48]. The results of this study reflect the coherence among content, function, and outcome as one of the crucial issues in MI intervention. Cumming and Williams [86] refer to this coherence as “meaningful imagery”, which describes the fit between what is imagined (what), the function (reason) that underlies it (why), and the output of the MI process. This coherence also illustrates the divergent function of MI as it facilitated the simultaneous development of BMS and S.C., although it was found to be partially not significant in S.C. From the social cognition perspective, the results of this study are related to the involvement of mirror neurons (MNs), a subpopulation of neurons that are active during the movement learning process, from observing movement and MI training to actual movement execution [87]. These MNs (possibly) link these three processes to social behavior. In the process, similar nervous systems are communal and share functions with each other, so that a series of movement learning processes is connected with the functional role of MNs to understand actions, intentions, imitation, and empathy in a social cognition mechanism [88]. Thus, according to these findings, the MI strategy has a simultaneous divergent function, as MI is effectively used to improve BMS and facilitate S.C. development. This is partly because of the commonality of the nervous system that is similar and shared in the MN system and a series of movement learning processes. Specifically, the involvement of MNs in MI training occurs when student-athletes perform image transformations by imagining what they will see when the object is manipulated to be more in line with the desired image.

The MI strategy also separately affected BMS mastery (Hypothesis 2a was supported). This finding can be explained partly by the presence of an equivalent nervous system communality during MI strategy training in actual movement training, with a lower magnitude [89]. This finding strengthens the brain activity hypothesis, which states that mental training (MI training) will be effective because it has similar neurophysiological activity between imagined movement and actual movement [83]. The four brain regions that are most consistently activated during MI training and actual training are the supplementary motor area, the premotor cortex, the parietal cortex, and the cerebellum [90]. Functionally, the neurophysiological communality of the two processes is complementary and mutually reinforcing, and both are involved in the overlapping brain system [91] to facilitate the representation of actual movements.

The results of this study were supported by the results of the MI fidelity check, which generally showed that youth beginner student-athletes perform MI effectively according to what they were taught. All fidelity check indicators showed above 75% achievement, except for the benefit indicator for S.C. development, which was only 70.70%. Furthermore, according to the simple effect interaction test results, C-MI had a higher effect than M-MI on BMS and S.C. mastery when combined with M-S.T. In contrast, M-MI had a higher effect than C-MI on S.C. when combined with I-S.T. Therefore, the use of C-MI and M-MI in the training process will be more effective when combined with M-S.T. and I-S.T., respectively.

Different results were found. Separately, S.T. and MI did not have a significant effect on S.C. (Hypotheses 1b and 2b were rejected). This finding is inconsistent with some of the previous study results [S.T., 26; MI: 48]. One of the causal descriptions related to the results of this study, among others, is that S.C. is a “fragile construct” [16] that is easily changeable, and its development is strongly influenced by success and failure in achieving goals; thus, it takes a relatively long time to form a robust S.C. Thus, S.T. and MI strategies can influence S.C. through indirect mechanisms, namely, through the accumulation of successes and failures in achieving goals. The S.C. level will be stable if there is an accumulation of goal achievement so that motivation becomes a steppingstone for S.C. development. From a methodological perspective, apart from the possibility that there was no initial measurement of the S.C. condition, considering that the indicators measured had to be directly related to the experience of the participants participating in badminton training, it was also due to the weakness of controlling the influence of psychosocial aspects to allow the diffusion of information and novelty effects.

### S.T. and MI interaction effect

The analysis results found that the combination of S.T. and MI strategies together had a significant interaction effect on the mastery of BMS and S.C. (Hypothesis 3 was supported). The results of this study complement the evidence on the effectiveness of a combination of S.T. and MI strategies in learning motor skills and sport psychology for beginner student-athlete participants when they are learning new motor skills, which is consistent with several previous studies [39, 92]. The results of this study explain, among others, the following: first, information in learning motor skills comes from verbal information channel systems (S.T.: action language imagination model [65] and nonverbal or movement observation and MI: dual coding model [62]). At the theoretical level, the two channels form a complementary process communality, where MI connects the motor system with the verbal system through the internal representation of the movement, while S.T. is used to generate image movements that will activate the internal representation of the movement. Longstaff [93]. calls these “mechanistic connections”; when the two information channels are used, student-athletes essentially transform verbal instructions into action, and vice versa. Second, there is a function communality at the applicative level based on S.T. and MI functions. Beginner student-athletes used S.T. and MI strategies for cognitive (instructional) and motivational functions whose outputs are functionally orthogonal use at the pedagogical level from the beginning to the end of the exercise. Thus, the combination of S.T. and MI strategies, whose functions are complementary and reinforcing and have an implicative effect on the S.C. development at the pedagogical level, is effective for increasing both the BMS and S.C. mastery because they functionally form a communality at the theoretical and applicable levels.

For the partial effect on S.C., the use of a combination of S.T. and MI in mastering BMS is a process that can activate the three self-efficacy antecedents, namely, past performance accomplishments, vicarious experience, and verbal persuasion [66]. The three antecedents function as cognitive strategies influencing the individual perception of self-efficacy and can increase self-efficacy through its influence on thought patterns and feelings of competence and success. In fact, these three antecedents strengthen the association between the mastery of BMS and of S.C. There is an additive-motivational effect of changes in BMS and an indication of a cyclical reciprocal relationship with S.C. by increasing the mechanism of the relationship between the two. Changes in one of the two variables are mutually determinant for changes in other variables, and vice versa.

Pairwise Comparison Test. A comparison of the effectiveness of I-S.T. and M-S.T. proved that I-S.T. had a higher effect on BMS (Hypothesis 4 was supported), even when it was compared to the CG. This finding is consistent with previous studies on child participants [20, 94] and provides partial support for the new matching hypothesis [32, 95], stating that I-S.T. is more effective than M-S.T. in the early stages of new advanced motor skill mastery, while the M-S.T. is more effective for learned motor tasks**.** This is partly because beginner student-athletes have a tendency to use movement as a medium for conducting internal dialog. In that regard, I-S.T. has more suitable content for internal dialog. I-S.T. is helpful for beginner student-athletes, especially because it is related to their limited time and attention capacity to process important information [83]. Within these limitations, I-S.T. can help increase the external and internal attention of beginner student-athletes to the relevant key elements of movement while restricting irrelevant stimuli. Therefore, attention is an inseparable constituent in the early stages of movement skill mastery (BMS).

The results of the pairwise comparisons test between C-MI and M-MI show that C-MI had a higher effect than M-MI on BMS mastery (Hypothesis 5 was supported). The results of this study are relevant to the findings of previous studies on beginner student-athlete participants [8, 43, 85, 96]. This is because C-MI activates and strengthens the neural network involved in motor execution, which is actually more equivalent and complementary than M-MI, which only imagines sociopsychological situations that support BMS mastery. As reported in several studies, M-MI is more profitable and effective for use in more emotionally demanding match situations [38]. C-MI plays a functional role as a coding system that can help beginner student-athletes master the symbolic aspects and movement patterns and create and develop a movement program by encoding the movement patterns to be carried out in the central nervous system, allowing them to practice the symbolic elements of the movement task they will perform [83, 97]. In other words, when using C-MI, student-athletes perform motor simulation exercises in their minds. Therefore, MI is actually a prototype form of motor simulation [98] and therefore can facilitate BMS mastery.

According to all the results of this study, the most important practical implication is that sports coaches and/or physical education teachers can use S.T. and MI strategies, either separately or jointly, to facilitate the motor learning, sports performance, and psychological skills of youth beginner student-athletes. The results of this study prove that youth beginner student-athletes can effectively use the S.T. and MI strategies when they are learning new motor skills (BMS) by completely adapting the structure of the learning process. The integration process must be carried out carefully using certain process stages [9]. Finally, for application, the use of ST should be combined with MI, I-S.T. with M-MI, and vice versa, while M-S.T. should be used with C-MI and vice versa. The results of this study prove that the combination at the functional level (S.T. and MI) and sub-functional level (I-S.T., M-S.T., C-MI M-MI) is more effective in improving BMS and S.C. mastery.

Several empirical findings show that there is a positive correlation between sports performance and S.C. [17, 99, 100]. In their research report, Hidayat and Budiman [17] interpret this relationship through a win‒win concept. The concept of a mutualistic relationship is based on the existence of a number of sources that influence motivation and S.C., namely, competency, level of ability, performance quality, experience, social support and incentives [101, 102]. According to this concept, the mastery of BMS is basically a manifestation of performance, and performance shows competency, level of ability, and performance quality. If the performance reaches the set target, then student athletes will receive social support and appreciation for their successful experience, which will increase their motivational aspects, such as motivation and self-confidence. Therefore, is the increased mastery of BMS has a motivational effect on S.C. This increase in the mastery of BMS led to an increase in S.C., and conversely, an increase in S.C. stimulated an increased mastery of BMS. The increase in both shows the interpretation of a cyclical reciprocal or bidirectional relationship.

Every study has limitations, including this research. The main limitations of this study are as follows: (1) the diversity of S.T. and MI strategies at the functional level (I-S.T., M-S.T., C-MI, M-MI) and the lack of differentiation at the sub-functional level. For this reason, the research results were not specific. (2) The observations were only based on the results of the posttest, and no pretest or retention test was carried out, and (3) the absence of a measure of participant imagery ability. Considering the three limitations of this study, further research needs to be conducted on the following topics: (1) the combination of S.T. and MI strategies by considering their elaboration to the sub-functional level as developed by Paivio [34] and Hall, et al. [35], (2) the research design should use a pretest–posttest design to better guarantee changes or provide counter factual information, (3) the involvement of imagery ability should be considered as one of the variables that mediates the effect of MI on appearance, as has been done in other studies [103–105], and (4) the involvement of the type of focus of attention, the type of motor skills, the skill level of the participants, and the use of neuro-physiological techniques should be considered to determine the structural and functional aspects of the brain that are activated during S.T. and MI so that the information obtained is more accurate, specific, and in depth,

## Conclusion

The findings of this study provide empirical evidence of the positive influence of the use of S.T. and MI, either separately or jointly, on the BMS and S.C. mastery of youth beginner student athletes, especially in the badminton training context. In particular, the results of this study can be generalized to individuals who have the same inclusive characteristics as the participants used in this study, including their setting in the training environment. This is because the concept of applying these two psychological strategies is designed for both male and female youth beginner student-athletes aged 10–12 years. These two psychological strategies can be incorporated into an integral part of the overall training program or be used by coaches and/or physical education teachers as alternative strategies to facilitate motor learning and sports performance and to develop psychological skills. This can be accomplished by completely adapting the structure of the learning process and integrating it into a model of the stages of the motor skills learning process from the perspective of social cognition.

## Data Availability

The datasets used and/or analyses during the current study are available from the corresponding author upon reasonable request.

## Abbreviations

S.T.: Self-talk
MI: Mental imagery
BMS: Badminton motor skills
S.C.: Self-confidence
M: Mean
SD: Standard deviation
EG: Experimental group
CG: Control group
I-S.T.: Instructional self-talk
M-S.T.: Motivational self-talk
C-MI: Cognitive mental imagery
M-MI: Motivational mental imagery
N: Number of participants
NG: Number of participants in a group
N_Ma_: Number of males
N_Fe_: Number of females
OBT: Outcome-based test PBT: process-based test
FL: Factor loading
SCS: Self-confidence scale
CE-D: Cognitive efficiency dimensions
PST-D: Physical skills and training dimensions
R-D: Resilience dimensions
